# Antileukemic Efficacy in Vitro of Talazoparib and APE1 Inhibitor III Combined with Decitabine in Myeloid Malignancies

**DOI:** 10.3390/cancers11101493

**Published:** 2019-10-03

**Authors:** Vanessa Kohl, Johanna Flach, Nicole Naumann, Susanne Brendel, Helga Kleiner, Christel Weiss, Wolfgang Seifarth, Daniel Nowak, Wolf-Karsten Hofmann, Alice Fabarius, Henning D. Popp

**Affiliations:** 1 Department of Hematology and Oncology, Medical Faculty Mannheim, Heidelberg University, 68167 Mannheim, Germany; Vanessa.Kohl@medma.uni-heidelberg.de (V.K.); Johanna.Flach@medma.uni-heidelberg.de (J.F.); Nicole.Naumann@medma.uni-heidelberg.de (N.N.); Susanne.Brendel@medma.uni-heidelberg.de (S.B.); Helga.Kleiner@medma.uni-heidelberg.de (H.K.); Wolfgang.Seifarth@medma.uni-heidelberg.de (W.S.); Daniel.Nowak@medma.uni-heidelberg.de (D.N.); w.k.hofmann@medma.uni-heidelberg.de (W.-K.H.); Alice.Fabarius@medma.uni-heidelberg.de (A.F.); 2 Department of Medical Statistics and Biomathematics, Medical Faculty Mannheim, Heidelberg University, 68167 Mannheim, Germany; Christel.Weiss@medma.uni-heidelberg.de

**Keywords:** myelodysplastic syndrome, chronic myelomonocytic leukemia, acute myeloid leukemia, talazoparib, APE1 inhibitor III, poly(ADP ribose) polymerase 1/2, apurinic/apyrimidinic endonuclease 1

## Abstract

Malignant hematopoietic cells of myelodysplastic syndromes (MDS)/chronic myelomonocytic leukemias (CMML) and acute myeloid leukemias (AML) may be vulnerable to inhibition of poly(ADP ribose) polymerase 1/2 (PARP1/2) and apurinic/apyrimidinic endonuclease 1 (APE1). PARP1/2 and APE1 are critical enzymes involved in single-strand break repair and base excision repair, respectively. Here, we investigated the cytotoxic efficacy of talazoparib and APE1 inhibitor III, inhibitors of PARP1/2 and APE1, in primary CD34+ MDS/CMML cell samples (*n* = 8; 4 MDS and 4 CMML) and in primary CD34+ or CD34− AML cell samples (*n* = 18) in comparison to healthy CD34+ donor cell samples (*n* = 8). Strikingly, talazoparib and APE1 inhibitor III demonstrated critical antileukemic efficacy in selected MDS/CMML and AML cell samples. Low doses of talazoparib and APE1 inhibitor III further increased the cytotoxic efficacy of decitabine in MDS/CMML and AML cells. Moreover, low doses of APE1 inhibitor III increased the cytotoxic efficacy of talazoparib in MDS/CMML and AML cells. In summary, talazoparib and APE1 inhibitor III demonstrated substantial antileukemic efficacy as single agents, in combination with decitabine, and combined with each other. Hence, our findings support further investigation of these agents in sophisticated clinical trials.

## 1. Introduction

Survival of patients belonging to the unfavorable risk groups of myelodysplastic syndromes (MDS)/chronic myelomonocytic leukemias (CMML) and acute myeloid leukemias (AML) is poor after standard antileukemic therapy [1,2,3,4,5]. Resistance to classical “7 + 3 chemotherapy” (cytarabine, daunorubicin) is common, and a substantial proportion of these patients is not eligible for allogeneic bone marrow transplantation. DNA methyltransferase inhibitors (DNMTi) (e.g., decitabine, azacytidine) may facilitate temporary remissions [6,7,8,9,10]; however, after failure to DNMTi, the median overall survival decreases to below six months [11,12,13,14].

Recent advances in the understanding of the biology of MDS/CMML and AML led to the development of novel targeted and untargeted agents (e.g., IDH inhibitors, FLT3 inhibitors, splicing modulators, novel DNMTi, anti-CD33 antibodies) [15,16,17,18,19,20]. In this context, inhibitors of poly(ADP-ribose) polymerase (PARP) 1/2 and apurinic/apyrimidinic endonuclease (APE) 1 may represent innovative DNA repair inhibitors that target MDS/CMML and AML cells by inhibition of single-strand break repair (SSB-R) and base excision repair (BER), respectively.

Talazoparib is a potent inhibitor of PARP1, which is involved in SSB-R and BER [21]. PARP1 senses and binds to single-strand breaks (SSBs) and synthesizes branched chains of poly(ADP-ribose) of up to several hundred units in length on itself and target proteins [22,23,24]. Talazoparib leads to the accumulation of SSBs, which may convert into DNA double-strand breaks (DSBs) eventually leading to cell death in DSB repair deficient cells [25,26]. In addition, talazoparib traps PARP1 at sites of damaged DNA and generates cytotoxic PARP1-DNA complexes, which may account for the main cytotoxic efficacy [27]. Recently, it has been demonstrated that low doses of DNMTi increase the trapping of PARP1 on DNA, which may enhance the killing of leukemic cells [28].

The APE1 inhibitor III inhibits APE1 [29], which is a key protein in BER [30]. After resection of the damaged base by glycosylases, APE1 cleaves the phosphodiester bound and creates a nick 5´ to the apurinic/apyrimidinic (AP) site. The AP site is then processed either by the short patch or the long patch BER pathway [31,32]. Inhibition of APE1 causes accumulation of AP sites, which block regular DNA synthesis [33]. As a result, stalled replication forks may collapse and convert into DSB [34], which then leads to death in DSB repair deficient leukemic cells [35].

Finally, 5-aza-2´-deoxycytidine (decitabine) is a potent DNMTi, which is used for the treatment of MDS/CMML [6,7,8] and AML [7,10]. Although the exact mechanism of action is not yet fully understood, it has been shown that decitabine modifies the epigenetic signature in MDS/CMML and AML cells and induces cell death by activation of tumor suppressor genes and silencing of oncogenes [36,37]. In addition, decitabine is misincorporated as a cytosine analogue during DNA replication and binds covalently DNMT1 to form cytotoxic DNMT1-DNA complexes [38].

In this study, we analyzed the antileukemic efficacy of talazoparib and APE1 inhibitor III in MDS/CMML and AML cells with defects in DSB repair. The cytotoxic efficacy of talazoparib and APE1 inhibitor III were tested alone, combined with decitabine, and combined with each other in primary CD34+ MDS/CMML cells and in primary CD34+ or CD34− AML cells in comparison to healthy CD34+ donor cells. We further analyzed the correlation of distinct parameters and biomarkers including cell proliferation, *PARP1*/*APE1* mRNA expression, *γ*H2AX foci levels, chromosomal aberrations, and gene mutations with cell survival.

## 2. Results

### 2.1. Cytotoxic Efficacy of Talazoparib and APE1 Inhibitor III in MDS/CMML and AML Cells

**Figure 1:**
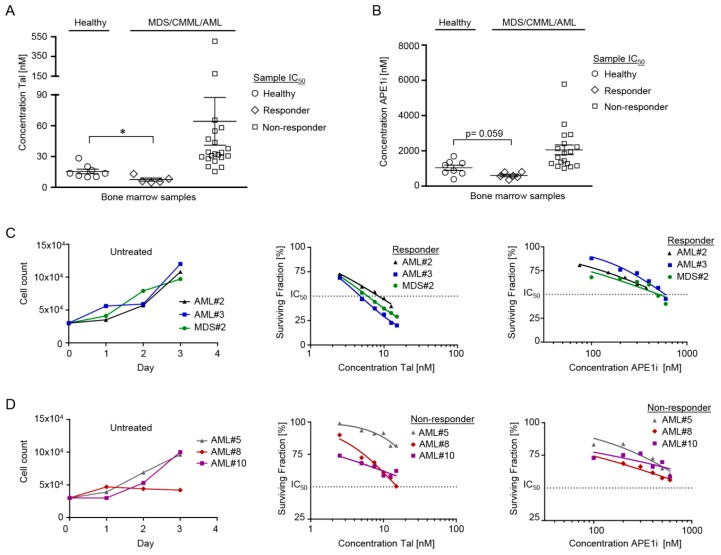
Cytotoxic efficacy of talazoparib and APE1 inhibitor III in healthy CD34+ donor cells, in CD34+ myelodysplastic syndrome (MDS)/chronic myelomonocytic leukemia (CMML) cells, and in CD34+ or CD34− acute myeloid leukemia (AML) cells after initial expansion for 3 days followed by 3 days of treatment. (**A**) The mean IC_50_ of talazoparib was significantly lower (* *p* = 0.016) in 1 MDS (MDS#2), 1 CMML (CMML#2), and 3 AML cell samples (AML#1, AML#2, AML#3) as compared to 8 healthy donor cell samples. (**B**) The mean IC_50_ of APE1 inhibitor III was substantially lower (*p* = 0.059) in 1 MDS (MDS#2) and 5 AML cell samples (AML#1, AML#2, AML#3, AML#6, AML#12) as compared to 8 healthy donor cell samples. (**C**) Exemplary growth curves (left panel) and corresponding surviving fractions of 3 ‘responders’ after initial expansion for 3 days followed by 3 days of treatment with talazoparib (mid panel) and APE1 inhibitor III (right panel). (**D**) Exemplary growth curves (left panel) and corresponding surviving fractions of 3 ‘non-responders’ after initial expansion for 3 days followed by 3 days of treatment with talazoparib (mid panel) and APE1 inhibitor III (right panel). Error bars represent mean ± standard error of mean.

The cytotoxic efficacy of talazoparib and APE1 inhibitor III was analyzed after 3 d of initial expansion followed by 3 d of treatment in CD34+ MDS/CMML cells (*n* = 8; 4 MDS and 4 CMML samples) and in CD34+ or CD34− AML cells (*n* = 18) in comparison to healthy CD34+ donor cells (*n* = 8) (Table 1, Figure 1). The comparison of IC_50_ values showed significantly increased (*p* = 0.016) cytotoxic efficacy of talazoparib in 2 MDS/CMML (MDS#2, CMML#2) and 3 AML cell samples (AML#1, AML#2, AML#3) (7 nM ± 2 (mean IC_50_ ± standard error of mean)) as compared to the 8 healthy donor cell samples (16 nM ± 2) (Figure 1A). The ’responder rate’ of MDS/CMML/AML samples towards talazoparib was about 19%. Furthermore, the cytotoxic efficacy of APE1 inhibitor III was substantially increased (*p* = 0.059) in 1 MDS (MDS#2) and 5 AML cell samples (AML#1, AML#2, AML#3, AML#6, AML#12) (603 nM ± 71) as compared to the cytotoxic efficacy in 8 healthy donor cell samples (1041 nM ± 149) (Figure 1B). The ’responder rate’ of MDS/CMML/AML samples towards APE1 inhibitor III was about 25%. Interestingly, 1 MDS (MDS#2) and 3 AML samples (AML#1-3) were ‘responders’ towards both talazoparib and APE1 inhibitor III.

The cell proliferation rate of MDS/CMML and AML cells might correlate with the cytotoxic efficacy of talazoparib and APE1 inhibitor III, respectively. Therefore, growth curves of untreated MDS/CMML and AML cells were correlated with the corresponding surviving fractions of talazoparib and APE1 inhibitor III treated MDS/CMML and AML cells (Figure 1C,D). However, no consistent correlation between cell proliferation and cytotoxic efficacy of talazoparib and APE1 inhibitor III was evident in MDS/CMML and AML cells. These findings suggest that the antileukemic efficacy of talazoparib and APE1 inhibitor III is not strictly dependent on the in vitro proliferation rate of leukemic blasts.

### 2.2. Cytotoxic Efficacy of Decitabine ± Talazoparib, Decitabine ± APE1 Inhibitor III, and Talazoparib ± APE1 Inhibitor III in MDS/CMML and AML Cells

**Figure 2:**
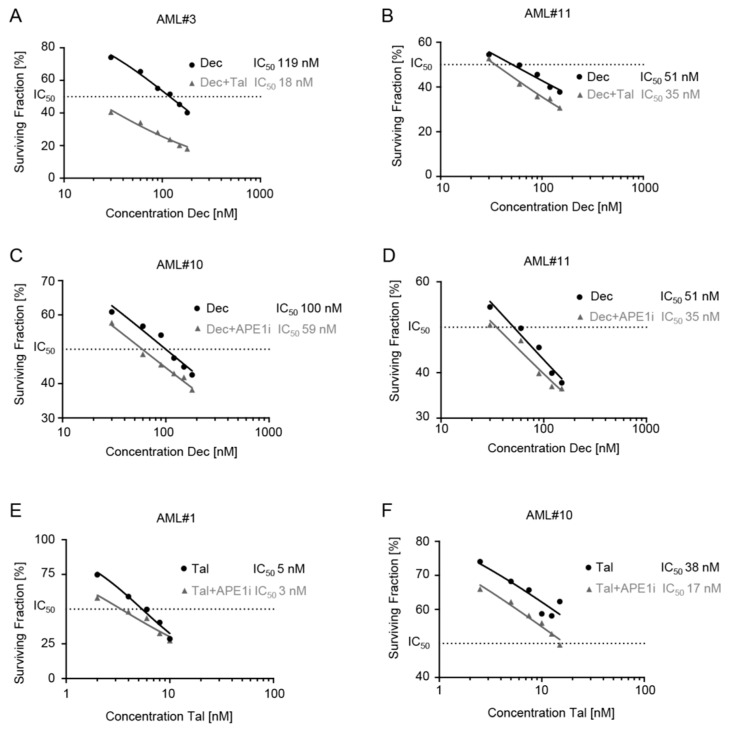
Surviving fractions of CD34+ or CD34− acute myeloid leukemia (AML) cells after initial expansion for 3 days followed by 3 days of treatment with (I) decitabine ± talazoparib, (II) decitabine ± APE1 inhibitor III, and (III) talazoparib ± APE1 inhibitor III. (**A**,**B**) Exemplary surviving fractions of AML cells treated with decitabine ± talazoparib. (**C**,**D**) Exemplary surviving fractions of AML cells treated with decitabine ± APE1 inhibitor III. (**E**,**F**) Exemplary surviving fractions of AML cells treated with talazoparib ± APE1 inhibitor III.

The cytotoxic efficacies of (I) decitabine ± talazoparib, (II) decitabine ± APE1 inhibitor III, and (III) talazoparib ± APE1 inhibitor III were analyzed in CD34+ MDS/CMML cells and in CD34+ or CD34− AML cells (Table 1, Figure 2). Subtoxic doses of talazoparib (2–2.5 nM) added to decitabine substantially decreased the surviving fraction in 4 MDS (MDS#1-4), 4 CMML (CMML#1–4), and 10 AML (AML#1, AML#3–6, AML#10, AML#11, AML#15–17) cell samples (Table 1, Figure 2A,B). Further, subtoxic doses of APE1 inhibitor III (50 nM) added to decitabine substantially decreased the surviving fraction in 1 MDS (MDS#3), 4 CMML (CMML#1–4), and 10 AML (AML#1, AML#3–5, AML#7, AML#8, AML#10, AML#11, AML#16, AML#17) cell samples (Table 1, Figure 2C,D). Finally, low subtoxic doses of APE1 inhibitor III (50 nM) added to talazoparib substantially decreased the surviving fraction in 2 MDS (MDS#2, MDS#3), 3 CMML (CMML#2–4), and 7 AML (AML#1, AML#3, AML#5, AML#7, AML#10, AML#11, AML#17) cell samples (Table 1, Figure 2E,F).

### 2.3. Analysis of PARP1 and APE1 mRNA Expression in MDS/CMML and AML Cells in Correlation to Cytotoxic Efficacy of Talazoparib and APE1 Inhibitor III

**Figure 3:**
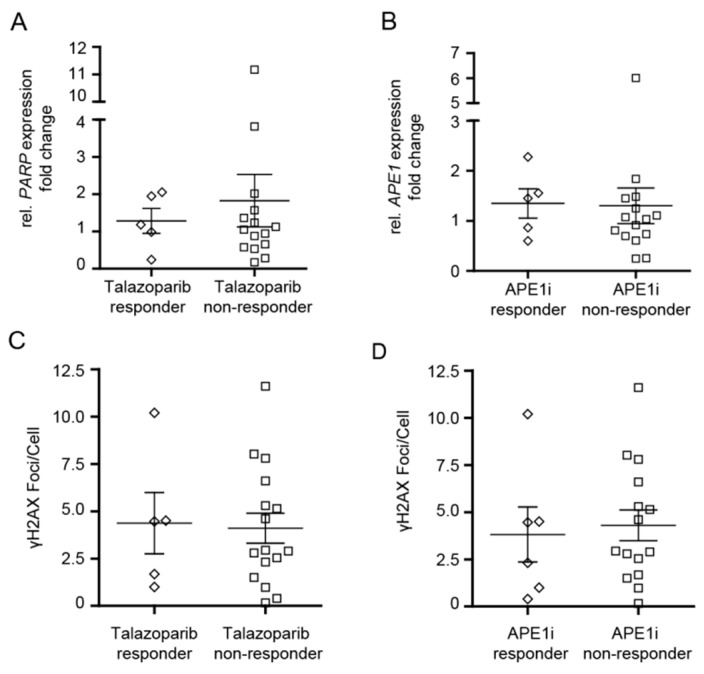
Cytotoxic efficacy of talazoparib and APE1 inhibitor III in CD34+ myelodysplastic syndrome (MDS)/chronic myelomonocytic leukemia (CMML) cells and in CD34+ or CD34− acute myeloid leukemia (AML) cells in relation to *PARP1*/*APE1* mRNA expression and *γ*H2AX foci levels. (**A**,**B**) *PARP1* and *APE1* mRNA expression levels in ‘responders’ and ‘non-responders’. (**C**,**D**) Levels of γH2AX foci in ‘responders’ and ‘non-responders’. Error bars represent mean ± standard error of mean.

*PARP1* and *APE1* mRNA expression might correlate with the cytotoxic efficacy of talazoparib and APE1 inhibitor III in MDS/CMML and AML cells and explain why some of the patients did not respond to the drugs. Therefore, *PARP1* and *APE1* mRNA expression levels were analyzed in MDS/CMML and AML cell samples. However, the *PARP1* and *APE1* mRNA expression levels did not differ significantly between ‘responders’ and ‘non-responders’ (Figure 3A,B).

### 2.4. Analysis of γH2AX Foci in MDS/CMML and AML Cells in Correlation to Cytotoxic Efficacy of Talazoparib and APE1 Inhibitor III

Since both drugs act on the DNA damage response, *γ*H2AX foci (as a readout for DNA damage) were analyzed for their potential correlation with the cytotoxic efficacy of talazoparib and APE1 inhibitor III in MDS/CMML and AML cells. Therefore, *γ*H2AX foci levels were quantified in MDS/CMML and AML cell samples. However, the number of *γ*H2AX foci did not significantly differ between ‘responders’ and ‘non-responders’ (Figure 3C,D).

### 2.5. Analysis of Chromosomal Aberrations and Gene Mutations in Correlation to Cytotoxic Efficacy of Talazoparib and APE1 Inhibitor III

Distinct chromosomal aberrations and gene mutations might correlate with the cytotoxic efficacy of talazoparib and APE1 inhibitor III in MDS/CMML and AML cells. Therefore, a comprehensive analysis of chromosomal aberrations and gene mutations was performed in MDS/CMML and AML cell samples (Table 2 and Table 3). Cytogenetic analysis revealed no specific chromosomal aberrations that predicted sensitivity towards talazoparib or APE1 inhibitor III. However, 100% of the talazoparib ‘responders’ (MDS#2, CMML#2, AML#1–3) and 83% of the APE1 inhibitor III ‘responders’ (MDS#2, AML#1–3, AML#6) displayed a normal karyotype. In addition, gene mutation analysis revealed that 50% of *KMT2A-PTD* mutated AML samples (AML#2, AML#3), 50% of *NRAS* mutated AML samples (AML#2, AML#3), and 67% of *U2AF1* mutated MDS/AML samples (MDS#2, AML#3) were sensitive towards talazoparib and APE1 inhibitor III, respectively. On the other hand, the AML sample (AML#12) most insensitive towards talazoparib (IC_50_ 505 nM) was *TP53-*mutated and harbored a complex karyotype. Further, the AML sample (AML#9) most insensitive towards APE1 inhibitor III (IC_50_ 5781 nM) was *DNMT3A-*, *FLT3-TKD-*, *IDH1-*, *NPM1-*, *NRAS-*mutated and harbored a normal karyotype. 

## 3. Discussion

In the present study, the cytotoxic efficacy of talazoparib and APE1 inhibitor III was investigated using several distinct approaches in CD34+ MDS/CMML cells and in CD34+ or CD34− AML cells in comparison to healthy CD34+ donor cells. In addition, potential predictive markers were analyzed and correlated with the cytotoxic efficacy of talazoparib and APE1 inhibitor III.

Talazoparib demonstrated critical antileukemic efficacy as a single-agent and in combination with decitabine in several MDS/CMML and AML cell samples, which might be mediated by the formation of cytotoxic PARP1-DNA complexes [27], DNMT1-DNA complexes [38], and PARP1-DNMT1-DNA complexes [28], respectively. Notably, our data suggest that decitabine combined with low doses of talazoparib might kill MDS/CMML and AML cells more efficiently than decitabine alone, as demonstrated by an increased antileukemic efficacy for the combination in about 86% of MDS/CMML/AML cell samples. Our observations confirm and extend results of previous experimental studies reporting on the antileukemic efficacy of olaparib [39,40,41] and talazoparib [28] and the synergistic efficacy between talazoparib and DNMTi in AML cell lines and primary AML cells [28]. Currently, the antileukemic efficacy of talazoparib alone and combined with decitabine has been tested in several clinical trials [42,43,44].

Furthermore, APE1 inhibitor III demonstrated antileukemic efficacy in several MDS/CMML and AML cell samples. The ‘responder’ rate of 25% in MDS/CMML/AML cell samples treated with APE1 inhibitor III was similar to the ‘responder’ rate of 19% in MDS/CMML/AML cell samples treated with talazoparib. However, APE1 inhibitor III exhibited about 30-fold less potent IC_50_ values as compared to talazoparib. Our observations are in accordance with the more advanced development of PARP inhibitors (e.g., talazoparib) as compared to APE1 inhibitors (e.g., APE1 inhibitor III). Despite the inferior potency of APE1 inhibitor III, subtoxic doses increased the antileukemic efficacy of decitabine and talazoparib in about 78% and 68% of MDS/CMML/AML cell samples, respectively. As BER is presumed to be involved in the repair of decitabine-induced DNA lesions [45], we hypothesize that APE1 inhibitor III might interact synergistically with decitabine by interruption of BER. Further, APE1 inhibitor III might interact synergistically with talazoparib by inducing suicidal cross-linking of PARP1 to AP sites [46].

Markers predicting the cytotoxic efficacy of talazoparib and APE1 inhibitor III in MDS/CMML and AML cells are, so far, largely unknown. Therefore, several markers including cell proliferation, PARP1/APE1 mRNA expression, *γ*H2AX foci levels, chromosomal aberrations, and gene mutations were analyzed and correlated with cell survival of MDS/CMML and AML cells after treatment with talazoparib and APE1 inhibitor III, respectively. Cell proliferation, *PARP1*/*APE1* mRNA expression, and *γ*H2AX foci levels demonstrated no consistent correlation with the cytotoxic efficacy of talazoparib and APE1 inhibitor III in MDS/CMML and AML cells. Furthermore, no specific chromosomal aberrations with a predictive impact were detected. However, 100% of the talazoparib ‘responders’ and 83% of the APE1 inhibitor III ‘responders’ displayed a normal karyotype. Finally, gene mutation analysis suggested that *KMT2A-PTD, NRAS,* and *U2AF1* mutations might be associated with a sensitivity rate of 50% or more towards talazoparib and APE1 inhibitor III, respectively.

Resistance to PARP inhibitors is a major clinical problem. Several categories of PARP inhibitor resistance mechanisms have been described to date and can be assigned to (I) increased drug efflux (e.g., upregulation of ABC transporters), (II) decreased trapping of PARP1 on DNA (e.g., trapping-diminishing PARP1 mutations), (III) restoration of homologous recombination (e.g., reactivation of *BRCA1/2*, loss of 53BP1, loss of Shieldin factors), and (IV) stabilization of stalled forks (e.g., loss of PTIP or EZH2) (reviewed in [47]). Further work is needed to clarify the role of these resistance mechanisms in MDS/CMML and AML. Possible mechanisms of resistance towards APE1 inhibitor III might include the expression of translesion polymerases (Pol) capable of bypassing AP sites or the upregulation of DSB repair genes.

## 4. Materials and Methods 

### 4.1. Bone Marrow Samples

This study was approved by the Ethics Committee II of the Medical Faculty of Mannheim at Heidelberg University (2018-566N-MA). Written, informed consent was obtained from all participants. Heparinized bone marrow (BM) samples were collected from 8 healthy donors (3 males, 5 females, mean age: 30 years), 8 MDS/CMML patients (4 MDS and 4 CMML; 5 males, 3 females, mean age: 70 years), and 18 AML patients (12 males, 6 females, mean age: 67 years) prior to antileukemic therapy (Table 1).

Mononuclear cells were separated from BM samples by Ficoll density gradient centrifugation. CD34+ cells were enriched using CD34 microbeads and cell separation columns (CD34 MicroBead Kit, Miltenyi Biotec, Bergisch Gladbach, Germany). Healthy CD34+ donor cells, CD34+ MDS/CMML cells, and CD34+ or CD34− AML cells were grown in a humidified 5% CO_2_ atmosphere at 37 °C in StemSpan medium (Stemcell Technologies Germany GmbH, Köln, Germany) supplemented with StemSpan myeloid expansion supplement (SCF, TPO, G-CSF, GM-CSF) (Stemcell Technologies Germany GmbH) and 1% penicillin/streptomycin.

### 4.2. Cytology, Flow Cytometry, Cytogenetics, and Gene Mutation Analysis

MDS/CMML and AML BM samples were diagnosed according to standard procedures using cytomorphology (May-Grünwald/Giemsa stained BM smears), flow cytometry, G-banding cytogenetics, fluorescence in situ hybridization (FISH), and molecular mutation analysis [48,49,50].

### 4.3. Cell Proliferation

Cell proliferation of untreated MDS/CMML and AML cells was determined by trypan blue exclusion assay at days 1, 2, and 3 [51].

### 4.4. Immunofluorescence Staining of γH2AX 

Immunofluorescence staining of *γ*H2AX was performed in untreated healthy donor cells, MDS/CMML cells, and AML cells using a mouse monoclonal anti-*γ*H2AX antibody (clone JBW301; Merck Millipore, Darmstadt, Germany) and an Alexa Fluor 488-conjugated goat anti-mouse secondary antibody (Thermo Fisher Scientific, Waltham, MA, USA) as previously described [52,53].

### 4.5. PARP1 and APE1 mRNA Expression

The mRNA expressions of *PARP1* and *APE1* were analyzed in healthy donor cells, MDS/CMML cells, and AML cells. For quantitative real-time PCR (qRT-PCR), the QuantiFast SYBR Green PCR Kit (Qiagen, Hilden, Germany) with commercial bioinformatically validated primer sets for *PARP1* (Hs_PARP1_1_SG QuantiTect Primer Assay, NM_001618) (Qiagen) and *APE1* (Hs_APEX1_1_SG QuantiTect Primer Assay, ENST00000216714) (Qiagen) was used according to manufacturer instructions. Normalization validation was performed with a primer set for the reference gene *GUSB* (Hs_GUSB_1_SG QuantiTect Primer Assay, ENST00000304895) (Qiagen). Samples were analyzed in triplicate using the LightCycler 480 II system (Roche, Mannheim, Germany) equipped with 96-well plates with a total well volume of 25 µL containing primer mix (2.5 µL), SYBR Green Master Mix (12.5 µL), 20 ng template cDNA (2 µL), and PCR-grade water (8 µL). qRT-PCR was performed at thermal cycling conditions of 95 °C for 5 min, 95 °C for 10 s, and 60 °C for 30 s (×40 cycles).

### 4.6. Drug Stock Solutions

Stock solutions of 50 mM talazoparib III (Selleck Chemicals, Houston, TX, USA), 5 mM APE1 inhibitor III (Selleck Chemicals), and 50 mM decitabine (Merck, Darmstadt, Germany) were prepared by dissolving the agents in dimethylsulfoxide (DMSO) (Merck).

### 4.7. Cell Survival Assay

The CellTiter-Glo luminescent cell viability assay (Promega, Southampton, UK) was used for the assessment of cell survival of healthy donor cells, MDS/CMML cells, and AML cells according to the manufacturer’s instructions. Cryopreserved CD34+ MDS/CMML cells and CD34+ or CD34− AML cells were thawed and expanded in culture for 3 d followed by 3 d of daily drug exposure. The IC_50_ designated the drug concentration capable of killing 50% of the cells and was calculated with GraphPad Prism 5 software (GraphPad Software, La Jolla, US) by linear regression. MDS/CMML and AML patient samples were classified as ‘responders’ or ‘non-responders’ using a cutoff value of mean IC_50_ of healthy controls minus standard error of mean. Experiments were performed once owing to the limited availability of healthy CD34+ donor cells, CD34+ MDS/CMML cells, and CD34+ or CD34− AML cells. DMSO-treated cells were used as control.

### 4.8. Statistical Analysis

All statistical calculations were done with SAS software, release 9.4 (SAS Institute Inc., Cary, NC, USA). For quantitative variables, mean values and standard errors were calculated. Categorical factors are presented with absolute and relative frequencies. In order to compare more than two groups, Kruskal–Wallis tests were performed. For pairwise comparisons, Wilcoxon two-sample tests were used. Test results with *p* values < 0.05 were considered as statistically significant.

For analysis of *PARP1* and *APE1* expressions, relative quantification was used according the ΔΔCT method. Normalization of target genes was performed using the arithmetic mean Ct of the nonregulated housekeeping gene *GUSB* from 6 healthy donor samples.

## 5. Conclusions

Our study performed on primary MDS/CMML and AML patient cells provides important preclinical data for a potential clinical use of talazoparib and APE1 inhibitor III alone and in combination with decitabine. In a next step, the in vivo antileukemic activities of talazoparib and APE1 inhibitor III need to be evaluated in clinical studies. Currently, talazoparib is tested as a single agent in advanced hematologic neoplasias (NCT01399840) [42] and in cohesin-mutated AML and MDS with excess blasts (NCT03974217) [43]. Further, decitabine and talazoparib are tested in patients with untreated and relapsed/refractory AML (NCT02878785) [44]. APE1 inhibitor III may enter similar trials in MDS/CMML/AML in the future.

## Figures and Tables

**Table 1:** Characterization of myelodysplastic syndrome/chronic myelomonocytic leukemia and acute myeloid leukemia bone marrow samples. APE1i: APE1 inhibitor III; CMML-0/1/2: chronic myeloid leukemia-0/1/2; Dec: decitabine; FISH: fluorescence in situ hybridization; IC_50_: half maximal inhibitory concentration; MDS-EB-1: myelodysplastic syndrome with excess blasts; MDS-MLD: myelodysplastic syndrome with multilineage dysplasia; sAML: secondary acute myeloid leukemia; Tal: talazoparib.

#	Age/Sex	Disease	Karyotype/FISH	Mutations	IC_50_ [nM]					
					Tal	APE1i	Dec	Dec + Tal	Dec + APE1i	Tal + APE1i
HEALTHY#1	25/♂	Healthy	46,XY[20]	-	20	1359	254	58	206	19
HEALTHY#2	23/♀	Healthy	46,XX[20]	-	28	392	23	-	-	-
HEALTHY#3	77/♀	Healthy	46,XX[20]	-	12	1195	156	52	128	10
HEALTHY#4	26/♂	Healthy	46,XY[20]	-	10	882	99	24	55	7
HEALTHY#5	21/♂	Healthy	46,XY[20]	-	14	724	113	14	102	8
HEALTHY#6	22/♀	Healthy	46,XX[20]	-	10	1697	111	26	111	10
HEALTHY#7	21/♀	Healthy	46,XX[20]	-	14	1305	147	35	123	12
HEALTHY#8	25/♀	Healthy	46,XX[20]	-	17	775	186	23	166	11
MDS#1	59/♂	MDS-EB-1	46,XY,-7,der(9)t(9;20)(p21;q13), der(20)t(9;20)(p21;q11),+der(20) t(9;20)(p21;q11)[17]/46,XY[3]	CBL, DNMT3A (VAR), EZH2 (VAR), FLT3-ITD	20	1357	160	48	137	19
MDS#2	73/♀	MDS-MLD	46,XX[20]	ASXL1, GATA2, RUNX1, U2AF1	6	509	9	3	8	5
MDS#3	68/ ♂	MDS-MLD	45,X,-Y[8]/45,X,-Y,del(1)(p34p36)[12]	ASXL1, U2AF1	26	1281	107	24	60	21
MDS#4	57♀	MDS-EB-1	46,XX[20]	ASXL1	31	2921	185	89	181	36
CMML#1	77/♂	CMML-1	46,XY,t(2;2)(p23;q32)[10]/47,XY, t(2;2)(p23;q32),+8[2]/46,XY[9]	ASXL1, CEBPA, IDH1, RUNX1, SRSF2	28	1095	42	25	32	28
CMML#2	73/♀	CMML-2	46,XX[20]	ASXL1, CEBPA, EZH2 (VAR), TET2	13	1286	210	62	159	11
CMML#3	84/♂	CMML-0	46,XY[20]	DNMT3A, RUNX1, SRSF2, TET2	175	1008	121	42	67	47
CMML#4	65/♂	CMML-2	46,XY[20]	ASXL1, IDH2, NRAS, PTPN11, ZRSR2	47	1954	153	48	122	35
AML#1	79/♂	sAML	46,XY[29]	ASXL1, FLT3-ITD, RUNX1, SF3B1	5	350	238	19	196	3
AML#2	63/♂	sAML	46,XY[25]	JAK2, KMT2A-PTD (MLL-PTD), NRAS, SRSF2, TET2 (VAR)	8	564	28	-	-	-
AML#3	76/♂	sAML	46,XY[17]	BCOR, DNMT3A, KMT2A-PTD (MLL-PTD), NRAS, TET2, U2AF1	5	593	119	18	100	4
AML#4	63/♀	*de novo* AML	46,XX,t(7;9)(q22;q34),add(17)(p12)[22]/46,XX[3]	ASXL1, DNMT3A, PTPN11, RUNX1	65	2407	172	93	137	84
AML#5	78/♂	sAML	47,XY,+8[3]/46,XY[17]	ASXL1, IDH2, SRSF2	31	1148	135	73	96	17
AML#6	83/♂	*de novo* AML	46,XY[20]	-	30	793	268	223	293	28
AML#7	72/♂	sAML	46,XY[20]	ASXL1, IDH2, SF3B1	44	1867	416	389	347	32
AML#8	70/♀	*de novo* AML	46,XX[20]	FLT3-ITD, NPM1, TET2	16	1122	91	92	64	15
AML#9	53/♀	*de novo* AML	46,XX[25]	DNMT3A, FLT3-TKD, IDH1, NPM1, NRAS	33	5781	348	763	642	85
AML#10	33/♀	*de novo* AML	46,XX[20]	DNMT3A (VAR), FLT3-ITD, KMT2A-PTD (MLL-PTD), KRAS	38	3511	100	46	59	17
AML#11	66/♂	*de novo* AML	47,XY,+8[13]/46,XY[7]	ASXL1, DNMT3A, IDH2, RUNX1, SRSF2	34	2893	51	34	35	25
AML#12	68/♀	*de novo* AML	51,XX,+1,der(2)t(2;12),der(5) t(5;13),+8, +11,der(12),-der(13),+15,+19,+mar[25]	TP53	505	808	215	-	231	-
AML#13	89/♂	sAML	47,XY,+8[23]/46,XY[2]	-	34	2131	205	294	318	119
AML#14	47/♂	*de novo* AML	42-46,XY,t(1;4)(p33;q35),del(3q),add(6q),-13, -15,-16,-17,-18,+19,+2-4mar [cp15]	DNMT3A, TP53	55	-	-	-	-	-
AML#15	63/♂	*de novo* AML	46,XY[11]	IDH2, NPM1, SRSF2	32	-	756	306	3077	40
AML#16	69/♂	sAML	47,XY,+21[6]/46,XY[14]	DNMT3A, KMT2A-PTD (MLL-PTD), RUNX1	19	1443	172	88	136	21
AML#17	69/♂	sAML	46,XY[26]	FLT3-ITD, GATA2, WT1	58	2226	248	145	181	32
AML#18	59/♀	*de novo* AML	45,XX[25]	BCOR, ETV6 (VAR), EZH2 (VAR), FLT3-ITD, NPM1, KRAS, TET2 (VAR)	29	1626	-	-	-	-

**Table 2:** Cytotoxic efficacy of talazoparib and APE1 inhibitor III in myelodysplastic syndromes/chronic myelomonocytic leukemias and acute myeloid leukemias in relation to chromosomal aberrations. AML: acute myeloid leukemia; CMML: chronic myelomonocytic leukemia; MDS: myelodysplastic syndrome.

	Talazoparib	APE1 inhibitor III	normal karyotype	-Y	-13	-15	-16	-17	-18	Trisomy 1	Monosomy 7	Trisomy 8	Trisomy 11	Trisomy 15	Trisomy 19	Trisomy 21	t(1;4)	t(2;2)	t(2;12)	t(5:13)	t(7;9)	t(9;20)	del(1)(p34p36)	del(3q)	del17p
AML#6		**Responder**																							
AML#12																									
MDS#2	**Responder**																								
AML#1																									
AML#2																									
AML#3																									
CMML#2		**Non-responder**																							
MDS#1	**Non-responder**																								
MDS#3																									
MDS#4																									
CMML#1																									
CMML#3																									
CMML#4																									
AML#4																									
AML#5																									
AML#7																									
AML#8																									
AML#9																									
AML#10																									
AML#11																									
AML#13																									
AML#14																									
AML#15																									
AML#16																									
AML#17																									
AML#18																									

**Table 3:** Cytotoxic efficacy of talazoparib and APE1 inhibitor III in myelodysplastic syndromes/chronic myelomonocytic leukemias and acute myeloid leukemias in relation to gene mutations. AML: acute myeloid leukemia; CMML: chronic myelomonocytic leukemia; MDS: myelodysplastic syndrome; VAR: variation.

	Talazoparib	APE1 inhibitor III	ASXL1	ASXL2	BCOR	CBL	CEBPA	DNMT3A	ETV6	EZH2	FLT3-ITD	FLT3-TKD	GATA1	GATA2	IDH1	IDH2	JAK2	KIT	KMT2A-PTD (MLL-PTD)	NPM1	NRAS	KRAS	PTPN11	RUNX1	SF3B1	SRSF2	TET2	TP53	U2AF1	WT1	ZRSR2
AML#6		**Responder**	not available																												
AML#12																															
MDS#2	**Responder**																														
AML#1																															
AML#2																											VAR				
AML#3																															
CMML#2		**Non-responder**								VAR																					
MDS#1	**Non-responder**							VAR		VAR																					
MDS#3																															
MDS#4																															
CMML#1																															
CMML#3																															
CMML#4																															
AML#4																															
AML#5																															
AML#7																															
AML#8																															
AML#9																															
AML#10								VAR																							
AML#11																															
AML#13			not available																												
AML#14																															
AML#15																															
AML#16																															
AML#17																															
AML#18									VAR	VAR																	VAR				

## References

[1] Shah A., Andersson T.M., Rachet B., Bjorkholm M., Lambert P.C. (2013). Survival and cure of acute myeloid leukaemia in England, 1971–2006: A population-based study. Br. J. Haematol..

[2] Greenberg P.L., Tuechler H., Schanz J., Sanz G., Garcia-Manero G., Sole F., Bennett J.M., Bowen D., Fenaux P., Dreyfus F. (2012). Revised international prognostic scoring system for myelodysplastic syndromes. Blood.

[3] Greenberg P., Cox C., LeBeau M.M., Fenaux P., Morel P., Sanz G., Sanz M., Vallespi T., Hamblin T., Oscier D. (1997). International scoring system for evaluating prognosis in myelodysplastic syndromes. Blood.

[4] Papaemmanuil E., Gerstung M., Bullinger L., Gaidzik V.I., Paschka P., Roberts N.D., Potter N.E., Heuser M., Thol F., Bolli N. (2016). Genomic Classification and Prognosis in Acute Myeloid Leukemia. N. Engl. J. Med..

[5] Patnaik M.M., Tefferi A. (2018). Chronic myelomonocytic leukemia: 2018 update on diagnosis, risk stratification and management. Am. J. Hematol..

[6] Lubbert M., Suciu S., Baila L., Ruter B.H., Platzbecker U., Giagounidis A., Selleslag D., Labar B., Germing U., Salih H.R. (2011). Low-dose decitabine versus best supportive care in elderly patients with intermediate- or high-risk myelodysplastic syndrome (MDS) ineligible for intensive chemotherapy: Final results of the randomized phase III study of the European Organisation for Research and Treatment of Cancer Leukemia Group and the German MDS Study Group. J. Clin. Oncol..

[7] Kantarjian H.M., Thomas X.G., Dmoszynska A., Wierzbowska A., Mazur G., Mayer J., Gau J.P., Chou W.C., Buckstein R., Cermak J. (2012). Multicenter, randomized, open-label, phase III trial of decitabine versus patient choice, with physician advice, of either supportive care or low-dose cytarabine for the treatment of older patients with newly diagnosed acute myeloid leukemia. J. Clin. Oncol..

[8] Kantarjian H., Issa J.P., Rosenfeld C.S., Bennett J.M., Albitar M., DiPersio J., Klimek V., Slack J., de Castro C., Ravandi F. (2006). Decitabine improves patient outcomes in myelodysplastic syndromes: Results of a phase III randomized study. Cancer.

[9] Fenaux P., Mufti G.J., Hellstrom-Lindberg E., Santini V., Finelli C., Giagounidis A., Schoch R., Gattermann N., Sanz G., List A. (2009). Efficacy of azacitidine compared with that of conventional care regimens in the treatment of higher-risk myelodysplastic syndromes: A randomised, open-label, phase III study. Lancet Oncol..

[10] Cashen A.F., Schiller G.J., O’Donnell M.R., DiPersio J.F. (2010). Multicenter, phase II study of decitabine for the first-line treatment of older patients with acute myeloid leukemia. J. Clin. Oncol..

[11] Jabbour E., Garcia-Manero G., Batty N., Shan J., O’Brien S., Cortes J., Ravandi F., Issa J.P., Kantarjian H. (2010). Outcome of patients with myelodysplastic syndrome after failure of decitabine therapy. Cancer.

[12] Prebet T., Gore S.D., Esterni B., Gardin C., Itzykson R., Thepot S., Dreyfus F., Rauzy O.B., Recher C., Ades L. (2011). Outcome of high-risk myelodysplastic syndrome after azacitidine treatment failure. J. Clin. Oncol..

[13] Prebet T., Gore S.D., Thepot S., Esterni B., Quesnel B., Beyne Rauzy O., Dreyfus F., Gardin C., Fenaux P., Vey N. (2012). Outcome of acute myeloid leukaemia following myelodysplastic syndrome after azacitidine treatment failure. Br. J. Haematol..

[14] Harel S., Cherait A., Berthon C., Willekens C., Park S., Rigal M., Brechignac S., Thepot S., Quesnel B., Gardin C. (2015). Outcome of patients with high risk Myelodysplastic Syndrome (MDS) and advanced Chronic Myelomonocytic Leukemia (CMML) treated with decitabine after azacitidine failure. Leuk. Res..

[15] Stone R.M., Mandrekar S.J., Sanford B.L., Laumann K., Geyer S., Bloomfield C.D., Thiede C., Prior T.W., Dohner K., Marcucci G. (2017). Midostaurin plus Chemotherapy for Acute Myeloid Leukemia with a FLT3 Mutation. N. Engl. J. Med..

[16] Stein E.M., DiNardo C.D., Pollyea D.A., Fathi A.T., Roboz G.J., Altman J.K., Stone R.M., DeAngelo D.J., Levine R.L., Flinn I.W. (2017). Enasidenib in mutant IDH2 relapsed or refractory acute myeloid leukemia. Blood.

[17] ClinicalTrials.gov (2018). An Open-label, Multicenter Phase 1 Trial to Evaluate the Safety, Pharmacokinetics and Pharmacodynamics of Splicing Modulator H3B-8800 for Subjects with Myelodysplastic Syndromes, Acute Myeloid Leukemia, and Chronic Myelomonocytic Leukemia. https://clinicaltrials.gov/ct2/show/NCT02841540.

[18] Issa J.J., Roboz G., Rizzieri D., Jabbour E., Stock W., O’Connell C., Yee K., Tibes R., Griffiths E.A., Walsh K. (2015). Safety and tolerability of guadecitabine (SGI-110) in patients with myelodysplastic syndrome and acute myeloid leukaemia: A multicentre, randomised, dose-escalation phase 1 study. Lancet Oncol..

[19] Kantarjian H.M., Roboz G.J., Kropf P.L., Yee K.W.L., O’Connell C.L., Tibes R., Walsh K.J., Podoltsev N.A., Griffiths E.A., Jabbour E. (2017). Guadecitabine (SGI-110) in treatment-naive patients with acute myeloid leukaemia: Phase 2 results from a multicentre, randomised, phase 1/2 trial. Lancet Oncol..

[20] Renneville A., Abdelali R.B., Chevret S., Nibourel O., Cheok M., Pautas C., Dulery R., Boyer T., Cayuela J.M., Hayette S. (2014). Clinical impact of gene mutations and lesions detected by SNP-array karyotyping in acute myeloid leukemia patients in the context of gemtuzumab ozogamicin treatment: Results of the ALFA-0701 trial. Oncotarget.

[21] Helleday T. (2011). The underlying mechanism for the PARP and BRCA synthetic lethality: Clearing up the misunderstandings. Mol. Oncol..

[22] Caldecott K.W. (2008). Single-strand break repair and genetic disease. Nat. Rev. Genet..

[23] Altmeyer M., Messner S., Hassa P.O., Fey M., Hottiger M.O. (2009). Molecular mechanism of poly(ADP-ribosyl)ation by PARP1 and identification of lysine residues as ADP-ribose acceptor sites. Nucleic Acids Res..

[24] Fahrer J., Kranaster R., Altmeyer M., Marx A., Burkle A. (2007). Quantitative analysis of the binding affinity of poly(ADP-ribose) to specific binding proteins as a function of chain length. Nucleic Acids Res..

[25] Shen Y., Rehman F.L., Feng Y., Boshuizen J., Bajrami I., Elliott R., Wang B., Lord C.J., Post L.E., Ashworth A. (2013). BMN 673, a novel and highly potent PARP1/2 inhibitor for the treatment of human cancers with DNA repair deficiency. Clin. Cancer Res..

[26] Curtin N. (2014). PARP inhibitors for anticancer therapy. Biochem. Soc. Trans..

[27] Murai J., Huang S.Y., Das B.B., Renaud A., Zhang Y., Doroshow J.H., Ji J., Takeda S., Pommier Y. (2012). Trapping of PARP1 and PARP2 by Clinical PARP Inhibitors. Cancer Res..

[28] Muvarak N.E., Chowdhury K., Xia L., Robert C., Choi E.Y., Cai Y., Bellani M., Zou Y., Singh Z.N., Duong V.H. (2016). Enhancing the Cytotoxic Effects of PARP Inhibitors with DNA Demethylating Agents—A Potential Therapy for Cancer. Cancer Cell.

[29] Rai G., Vyjayanti V.N., Dorjsuren D., Simeonov A., Jadhav A., Wilson D.M., Maloney D.J. (2012). Synthesis, biological evaluation, and structure-activity relationships of a novel class of apurinic/apyrimidinic endonuclease 1 inhibitors. J. Med. Chem..

[30] Lindahl T. (1979). DNA glycosylases, endonucleases for apurinic/apyrimidinic sites, and base excision-repair. Prog. Nucleic Acid Res. Mol. Biol..

[31] Robertson A.B., Klungland A., Rognes T., Leiros I. (2009). DNA repair in mammalian cells: Base excision repair: The long and short of it. Cell. Mol. Life Sci..

[32] Krokan H.E., Bjoras M. (2013). Base excision repair. Cold Spring Harb. Perspect. Biol..

[33] Jain R., Aggarwal A.K., Rechkoblit O. (2018). Eukaryotic DNA polymerases. Curr. Opin. Struct. Biol..

[34] Yeeles J.T., Poli J., Marians K.J., Pasero P. (2013). Rescuing stalled or damaged replication forks. Cold Spring Harb. Perspect. Biol..

[35] Sultana R., McNeill D.R., Abbotts R., Mohammed M.Z., Zdzienicka M.Z., Qutob H., Seedhouse C., Laughton C.A., Fischer P.M., Patel P.M. (2012). Synthetic lethal targeting of DNA double-strand break repair deficient cells by human apurinic/apyrimidinic endonuclease inhibitors. Int. J. Cancer.

[36] Baylin S.B., Jones P.A. (2011). A decade of exploring the cancer epigenome—Biological and translational implications. Nat. Rev. Cancer.

[37] Issa J.P. (2007). DNA methylation as a therapeutic target in cancer. Clin. Cancer Res..

[38] Patel K., Dickson J., Din S., Macleod K., Jodrell D., Ramsahoye B. (2010). Targeting of 5-aza-2’-deoxycytidine residues by chromatin-associated DNMT1 induces proteasomal degradation of the free enzyme. Nucleic Acids Res..

[39] Esposito M.T., Zhao L., Fung T.K., Rane J.K., Wilson A., Martin N., Gil J., Leung A.Y., Ashworth A., So C.W. (2015). Synthetic lethal targeting of oncogenic transcription factors in acute leukemia by PARP inhibitors. Nat. Med..

[40] Gaymes T.J., Shall S., MacPherson L.J., Twine N.A., Lea N.C., Farzaneh F., Mufti G.J. (2009). Inhibitors of poly ADP-ribose polymerase (PARP) induce apoptosis of myeloid leukemic cells: Potential for therapy of myeloid leukemia and myelodysplastic syndromes. Haematologica.

[41] Faraoni I., Compagnone M., Lavorgna S., Angelini D.F., Cencioni M.T., Piras E., Panetta P., Ottone T., Dolci S., Venditti A. (2015). BRCA1, PARP1 and gammaH2AX in acute myeloid leukemia: Role as biomarkers of response to the PARP inhibitor olaparib. Biochim. Biophys. Acta.

[42] ClinicalTrials.gov Study of BMN 673, a PARP Inhibitor, in Patients with Advanced Hematological Malignancies. https://clinicaltrials.gov/ct2/show/NCT01399840?term=BMN+673%2C+a+PARP+inhibitor&rank=2.

[43] ClinicalTrials.gov Talazoparib for Cohesin-Mutated AML and MDS With Excess Blasts. https://clinicaltrials.gov/ct2/show/NCT03974217?term=talazoparib%2C+cohesin&rank=1.

[44] ClinicalTrials.gov Decitabine and Talazoparib in Untreated AML and R/R AML (1565GCC). https://clinicaltrials.gov/ct2/show/NCT02878785?term=Decitabine+and+Talazoparib+in+Untreated+AML+and+R%2FR+AML&rank=1.

[45] Orta M.L., Hoglund A., Calderon-Montano J.M., Dominguez I., Burgos-Moron E., Visnes T., Pastor N., Strom C., Lopez-lazaro M., Helleday T. (2014). The PARP inhibitor Olaparib disrupts base excision repair of 5-aza-2’-deoxycytidine lesions. Nucleic Acids Res..

[46] Prasad R., Horton J.K., Chastain P.D., Gassman N.R., Freudenthal B.D., Hou E.W., Wilson S.H. (2014). Suicidal cross-linking of PARP-1 to AP site intermediates in cells undergoing base excision repair. Nucleic Acids Res..

[47] Noordermeer S.M., van Attikum H. (2019). PARP Inhibitor Resistance: A Tug-of-War in BRCA-Mutated Cells. Trends Cell Biol..

[48] Löffler H., Rastetter J., Haferlach T. (2005). Light microscopic procedures. Atlas of Clinical Hematology.

[49] Gisselsson D., Heim S., Mitelman F. (2009). Cytogenetic methods. Cancer Cytogenetics.

[50] MLL Request for Testing Form. https://www.mll.com/en.html.

[51] Strober W. (2015). Trypan Blue Exclusion Test of Cell Viability. Curr. Protoc. Immunol..

[52] Popp H.D., Brendel S., Hofmann W.K., Fabarius A. (2017). Immunofluorescence Microscopy of γH2AX and 53BP1 for Analyzing the Formation and Repair of DNA Double-strand Breaks. J. Vis. Exp..

[53] Popp H.D., Naumann N., Brendel S., Henzler T., Weiss C., Hofmann W.K., Fabarius A. (2017). Increase of DNA damage and alteration of the DNA damage response in myelodysplastic syndromes and acute myeloid leukemias. Leuk. Res..

